# Parathyroid Hormone Causes Endothelial Dysfunction by Inducing Mitochondrial ROS and Specific Oxidative Signal Transduction Modifications

**DOI:** 10.1155/2018/9582319

**Published:** 2018-12-19

**Authors:** Jessica Gambardella, Matteo De Rosa, Daniela Sorriento, Nella Prevete, Antonella Fiordelisi, Michele Ciccarelli, Bruno Trimarco, Nicola De Luca, Guido Iaccarino

**Affiliations:** ^1^Dept. of Advanced Biomedical Sciences, Federico II University, Napoli, Italy; ^2^Dept. of Translational Medical Sciences, Federico II University, Napoli, Italy; ^3^Institute of Endocrinology and Experimental Oncology (IEOS), CNR, Napoli, Italy; ^4^Dept. of Medicine, Surgery and Dentistry, University of Salerno, Italy

## Abstract

Vitamin D deficiency contributes to cardiovascular risk (CVR), with hyperparathyroidism advocated as a putative mechanism. Indeed, mounting evidence supports the hypothesis that parathyroid hormone (PTH) impairs endothelial function, even though mechanisms are not fully elucidated. The present study was designed to verify *in vitro* the ability of sustained exposure to PTH to cause endothelial dysfunction, exploring the underlying mechanisms. In bovine aortic endothelial cells (BAECs), we evaluated the effects of PTH exposure (0.1 nM–24 hours) on both endothelial response to vasodilators, such as bradykinin (Bk (30 nM)) and acetylcholine (Ach (1 *μ*M)), and angiogenic competence. Pretreatment with PTH impaired endothelial response to Bk but not to Ach, in terms of cytosolic calcium fluxes and NO production. In order to explore the underlying mechanisms, we assessed the production of total and mitochondrial ROS (tROS and mROS, respectively) in response to PTH (at 1 and 3 hours). PTH increased ROS generation, to an extent high enough to determine oxidation of Bk receptor B2. Conversely, the oxidation levels of M1 and M3 Ach receptors were not affected by PTH. A mROS selective scavenger (MitoTEMPO (5 *μ*M)) restored the endothelial responsiveness to Bk while the well-known antioxidant properties of vitamin D (100 nM) failed to counteract PTH-mediated oxidative stress. PTH determined mitochondrial calcium fluxes ([Ca^2+^]_mt_) and the mitochondrial calcium uniporter inhibitor Ru360 (10 *μ*M) reduced mROS production and prevented the PTH-mediated endothelial dysfunction. Angiogenic competence was evaluated as tubular formations in the endothelial Matrigel assay and showed a significant impairment in PTH-pretreated cells (0.1 nM–24 hours), despite the increase in VEGF transcriptional levels. VEGFR2 oxidation occurred in response to PTH, suggesting that even the impairment of angiogenesis was due to the ROS surge. These results indicate that PTH affects endothelial function through ROS production, driven by mitochondrial calcium overload. PTH-induced oxidative stress might act as signaling modifiers, altering specific pathways (Bk and VEGF) and preserving others (Ach).

## 1. Background

Parathyroid hormone (PTH) is a polypeptide consisting of 84 amino acids, whose synthesis, maturation, and secretion occur at the level of parathyroid glands. The main physiological role of this hormone is the reciprocal fine regulation of serum Ca^2+^ concentrations [1]. Lowering levels of serum Ca^2+^ trigger PTH secretion which in turn increases intestinal Ca^2+^ absorption, reduces renal Ca^2+^ excretion, and promotes the release of Ca^2+^ from the bone, thus restoring the physiological serum levels [1]. Feedback inhibition of PTH secretion is reciprocally mediated by the increase of serum Ca^2+^ levels. A mechanism of further complexity is the PTH-mediated activation of calcidiol (25-hydroxyvitamin D) in calcitriol (1,25-dihydroxyvitamin D) in renal tubular cells: calcitriol in turn inhibits PTH secretion directly through a receptor-mediated mechanism that reduces the PTH synthesis rate [2]. Furthermore, calcitriol facilitation of intestinal Ca^2+^ adsorption leads to the inhibition of PTH secretion, through the induced increase in serum Ca^2+^ levels.

The pleiotropic effects of PTH are mediated by a single receptor, the PTH1 receptor (PTH1R) [3]. PTH1R, a member of the G protein-coupled receptor (GPCR) family, can couple to either Gs or Gq depending on a tissue-type specificity. This receptor is almost ubiquitous, with higher expression in the bone and kidney [3]. In addition, the PTH1R is also observed into the myocardium and vasculature, with potential implication in cardiovascular diseases. Indeed, PTH has been lately considered a mediator for bone-renal-vascular interactions and a novel causative factor for the development of cardiovascular disorders, including hypertension [4, 5], left ventricular hypertrophy [6], congestive heart failure (HF) [7, 8], and both fatal and nonfatal cardiovascular events [9, 10]. Moreover, higher serum concentrations of PTH correlate with worse prognosis in the setting of cardiovascular diseases, and above-physiological PTH levels independently predict higher risk of hospitalizations, cardiovascular events, and all-cause mortality in HF or stable coronary artery disease [11, 12]. Interestingly, even in individuals with PTH within the normal range, higher plasma levels of PTH associate with higher cardiovascular risk (CVR), independent of other confounding cardiovascular risk factors [13, 14]. Several mechanisms have been advocated to explain the link between PTH and CVR. Indeed, PTH has been directly implicated in a wide range of vascular alterations, such as endothelial dysfunction, vascular calcification, and vascular remodeling, which lead to atherogenesis and arterial stiffness [15–19]. The relative contribution of vascular alterations in PTH-induced increased CVR is still debated, but mounting evidence supports the pivotal role of endothelial dysfunction, even in the early phases of chronic exposure to higher PTH levels. However, how PTH negatively modulates endothelial function is a still unsolved question.

Several mechanisms contribute to endothelial dysfunction, and among these, the oxidative stress seems to have a determinant role; indeed, sustained ROS production can inactivate nitric oxide (NO), thus contributing to impaired endothelial-dependent vasodilatation [20]. The potential regulation of ROS production by PTH has been hypothesized [21], since after parathyroidectomy in patients, the decrease of oxidative stress markers occurs [22, 23]. However, a mechanistic link between PTH and altered redox balance is still to be verified.

The present study was designed to verify *in vitro* the ability of sustained exposure to PTH to cause endothelial dysfunction, with a particular focus on the potential role of oxidative stress.

## 2. Materials and Methods

### 2.1. Cell Cultures

*In vitro* studies were performed on cell cultures of bovine aortic endothelial cells (BAECs), cultured in Roswell Park Memorial Institute (RPMI) 1640 medium supplemented with 10% fetal bovine serum (FBS) at standard conditions of 37°C in 95% air and 5% CO_2_. All experiments were performed in triplicate with cells between passages 5 and 9.

### 2.2. Cytosolic and Mitochondrial Calcium Determinations

Cytosolic Ca^2+^ levels were detected in BAECs using Fluo4-AM dye (Invitrogen). Cells seeded in 24-well plates were incubated with 5 *μ*M Fluo4-AM for 45 min at 37°C and 15 min at room temperature. After the incubation time, cells were washed twice with PBS, and fresh medium was added. Raw fluorescence values at 485/530 nm were collected every 10 sec for 5 min at 37°C using a microplate fluorescence reader (Tecan Infinite 200 Pro). After 10 sec of basal line measurement, 30 nM bradykinin, 1 *μ*M acetylcholine, or 0.1 nM PTH was automatically injected. The fluorescent signal was corrected for the background signal derived from nonmarked cells. Same experimental conditions were maintained to assess mitochondrial calcium uptake using Rhod-2 AM dye (Molecular Probes). BAECs were incubated with 5 *μ*M Rhod-2 AM for 30 min at 37°C. After the incubation time, cells were abundantly washed with indicator-free medium to remove any dye that is nonspecifically associated with the cell surface and then incubated for further 45 min to allow the complete de-esterification of intracellular AM dye and the spontaneous elimination of cytosolic staining, whereas mitochondrial staining was retained. After this second step of incubation, raw fluorescence values at 530/580 nm were collected every 10 sec, and at the second kinetic cycle, PTH 0.1 nM was automatically injected. The fluorescence was corrected by the background signal derived from nonmarked cells. All data were reported as *F*–*F*_0_ (*F* = fluorescence signal of BAECs stimulated with either Bk, Ach, or PTH; *F*_0_ = fluorescence signal of unstimulated BAECs).

### 2.3. Determination of NO Production

BAECs were seeded in 24-well plates and incubated with 10 *μ*M DAF-FM Diacetate for 60 min at 37°C (Invitrogen) to evaluate NO production levels. After washing to remove excess probe, the cells were incubated for additional 15 min to allow complete de-esterification of internalized probe. Raw fluorescence at 495/515 nm was registered every 10 sec using the Tecan Infinite 200 Pro plate reader. After 10 sec of basal line measurement, either 30 nM bradykinin or 1 *μ*M acetylcholine was automatically injected. The fluorescence was corrected by the background signal derived from nonmarked cells. Data were reported as *F*–*F*_0_ (*F* = fluorescence signal of BAECs stimulated with either Bk or Ach; *F*_0_ = fluorescence signal of unstimulated BAECs).

### 2.4. Evaluation of Total and Mitochondrial ROS Productions

BAECs were plated at density of 5 × 10^4^ for each well in 24-well plates. After incubation with PTH 0.1 nM for both 1 and 3 h, the cells were incubated with either 5 *μ*M H_2_DCFDA (Invitrogen) for 30 min at 37°C for total ROS (tROS) detection or with 5 *μ*M Mitosox (Invitrogen) for 10 min at 37°C for mitochondrial ROS (mROS) detection, in a humidified atmosphere (5% CO_2_, 95% air). After incubation, cells were washed twice with PBS, and fresh medium was added. The fluorescence was immediately measured by a plate reader (Tecan Infinite 200 Pro) using excitation/emission wavelengths of 492/520 nm for H_2_DCFDA and 510/580 nm for Mitosox. Then, the cells were trypsinized and collected for cytofluorimetric analysis. In some experiments, cells were also treated with either 5 *μ*M MitoTEMPO (Sigma-Aldrich), 100 nM calcipotriol hydrate, a vitamin D analogue (Sigma-Aldrich), or 10 *μ*M Ru360 (Merck-Millipore). MitoTEMPO was administered 30 min before PTH, while both calcipotriol hydrate and Ru360 were coadministered with PTH.

### 2.5. Cytofluorimetry

H_2_DCFDA and Mitosox-loaded BAECs were analyzed by flow cytometry (FACSCalibur, BD Biosciences) followed by analysis of mean fluorescence intensity of 10,000 events by Cellquest software (BD Biosciences).

### 2.6. Induction of Cell Hypoxia

BAECs were treated with a hypoxia-specific medium containing (mM) concentrations of 116 NaCl, 5.4 KCl, 0.8 MgSO_4_, 26.2 NaHCO_3_, 1 NaH_2_PO_4_, 1.8 CaCl_2_, 0.01 glycine, and 0.001 (% *w*/*v*) phenol red. Before the addition to cells, this medium was saturated for 10 min at 1 atm with 95% N_2_ and 5% CO_2_ mixture; the cells in the described medium were incubated in an anaerobic chamber (hypoxia chamber) filled with the same gas mixture, at 37°C. The pH, pO_2,_ and pCO_2_ of the resulting medium were 7.36 ± 0.2, 45.3 ± 1.2 mmHg, and 35.3 ± 0.8 mmHg and 7.32 ± 0.9, 32.6 ± 1.1, and 37.9 ± 2.1 mmHg, before and at the end of hypoxia, respectively. After 1 h of incubation in hypoxia condition, the cells were used for different experimental determinations.

### 2.7. Immunoprecipitation

Cells were lysed in RIPA/SDS buffer [50 mM Tris-HCl (pH 7.5), 150 mM NaCl, 1% Nonidet P-40, 0.25% deoxycholate, 9.4 mg/50 ml sodium orthovanadate, 20% SDS]. Protein concentration was determined by using the BCA assay kit (Pierce). Endogenous bradykinin receptor 2 (B2), muscarinic receptors 1 and 3 (M1 and M3), and vascular endothelial growth factor receptor 2 (VEGFR2) from total extracts were immunoprecipitated with specific antibodies (Santa Cruz for B2, M1, and M3 and Cell Signaling for VEGFR2) and protein A/G agarose (Santa Cruz), incubated at 4°C overnight. After centrifugation and extensive washes, the immunocomplexes were isolated; through the addition of 2% SDS and incubation at 95°C, denaturation of immunocomplexes was performed allowing to remove the agarose beads. The protein concentration of solution containing the immunoprecipitated receptors was measured to ensure the use of the same protein quantity.

### 2.8. Derivatization of the Carbonyl Group for Detecting Protein Oxidation and Western Blot Analysis

The detection of carbonyl groups introduced into proteins by oxidative reactions occurring inside cells was performed using OxyBlot Protein Oxidation Detection Kit (Millipore) by following the manufacture protocol. Briefly, 5 *μ*l containing 15 *μ*g of proteins from cell lysate or from immunoprecipitated preparation was derivatized through reaction with 2,4-dinitrophenylhydrazine (DNPH), which converts the carbonyl groups of proteins in 2,4-dinitrophenylhydrazone (DNP-hydrazone). The DNP-derivatized protein mixtures were analyzed by Western Blot. Briefly, proteins were separated by 4–12% SDS/PAGE gel and transferred to an Immobilon-P nitrocellulose filter (Millipore Corporation); the membranes were blocked in Tris-buffered saline containing 0.002 g/l Tween 20 (TBST) and 0.05 g/l nonfat dry milk. After blocking, the membranes were washed three times with TBST and then incubated overnight at 4°C in 5% BSA TBST with a primary specific antibody: DNP levels were visualized by a specific primary and secondary antibody provided by the oxidation kit while the levels of immunoprecipitated B2, M1, M3, and VEGFR2 were visualized through the same specific antibody used for their immunoprecipitation. The whole lysate was used as the positive control. As the negative controls, the assay was performed using a nonspecific antibody from the same species as the IP antibody. A standard chemiluminescence reaction (PIERCE) was used to produce a radiographic signal. Blots from 3 independent experiments were quantified and corrected for an appropriate loading control. Densitometric analysis was performed using Image Quant software (Molecular Dynamics Inc.).

### 2.9. *In Vitro* Matrigel Angiogenesis Assay

The formation of network-like structures by BAECs on an extracellular matrix- (ECM-) like 3D gel consisting of Matrigel® (BD Biosciences, Bedford, MA, USA) was performed as previously described and validated [24]. The six-well multidishes were coated with growth factor-reduced Matrigel according to the manufacturer's instructions. BAECs (5 × 10^4^) were seeded on Matrigel Matrix in the absence (CTRL) or the presence of PTH (0.1 nM) and incubated at 37°C for 24 h. After incubation, BAECs underwent differentiation into capillary-like tube structures. Tubule formation was defined as a structure exhibiting a length four times its width [24]. Network formation was observed using an inverted phase contrast microscope (Zeiss). Representative fields were taken, and the average of the total number of brunch points was counted in 15 random fields by two independent investigators.

### 2.10. Real-Time PCR

Total RNA from BAECs was extracted using a TRIzol reagent (Invitrogen), and cDNA was synthetized by means of ThermoScript RT-PCR System (Invitrogen), following the manufacturer instruction. After reverse transcription reaction, real-time quantitative polymerase chain reaction (RT-PCR) was performed with the SYBR Green real-time PCR master mix kit (Applied Biosystems, Foster City, CA, USA) as described [25]. The reaction was visualized by SYBR Green-derived fluorescence analysis on StepOne instrument (Applied Biosystem). Primers for gene analysis were as follows: 18S: For 5′GTAACCCGTTGAACCCATT3′, Rev 5′CCATCCAATCGGTAG-TAGCG3′ and VEGF2A: For 5′CAGGCTGTCGTAACGATGAA3′, Rev 5′TTTCTTGCGCTTTCGTTTTT3′.

All standards and samples were assayed in triplicate. Thermal cycling was initiated with an initial denaturation at 95°C for 5 min. After this initial step, 40 cycles of PCR were performed. Each RT-PCR cycle consisted of heating at 95°C for 15 seconds for melting, 60°C for 30 seconds for annealing, and 72°C for 1 minute for the extension. The ratio of fold change was calculated using the Pfaffl method [26].

### 2.11. Statistical Analysis

Slopes and elevations of both cytosolic Ca^2+^ and NO kinetics were compared between all experimental groups. Both the peak of Ca^2+^ increase and NO release and plateau amplitude of Ca^2+^ kinetics of all experimental groups were compared according to paired *t*-test or one-way ANOVA to assess statistical significance. One-way ANOVA was also performed to compare tROS and mROS levels among the different groups. Bonferroni's multiple comparison test was then performed where applicable. Unpaired *t*-test was performed to compare among experimental groups both levels of protein oxidation vs. CTRL, VEGF transcriptional levels, and the number of branch points in the endothelial Matrigel assay. A significance level of *p* < 0.05 was assumed for all statistical evaluations. Statistics were computed with GraphPad Prism software (San Diego, California).

## 3. Results

### 3.1. PTH Impairs Endothelial Function through Selective Interference on Bradykinin Signaling

**Figure 1:**
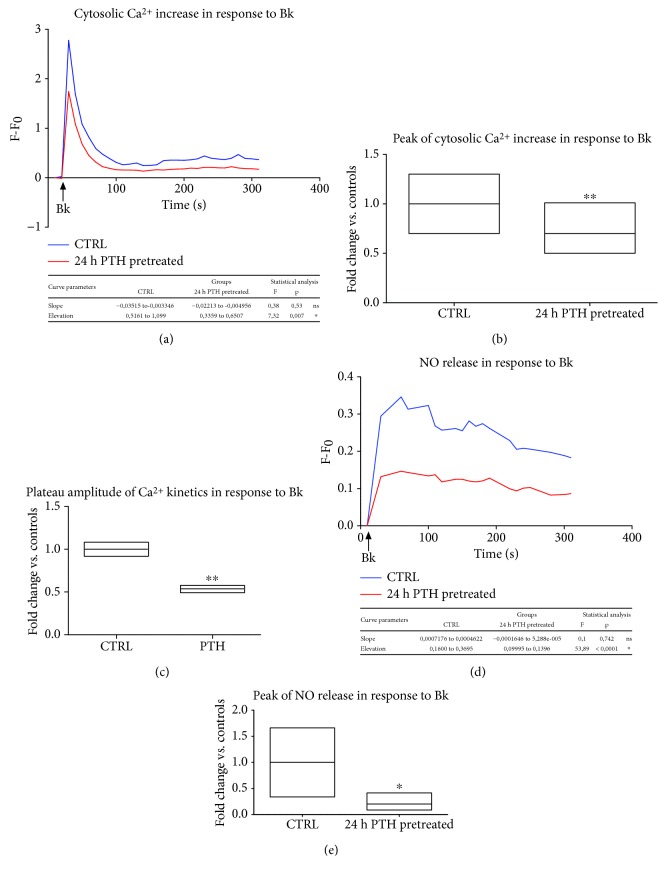
PTH impairs endothelial responses to bradykinin (Bk) (a–e). BAECs were treated with PTH (0.1 nM) for 24 hours, and Ca^2+^ fluxes were assessed in response to Bk (30 nM), incubating the cells with the fluorescent probe Fluo4-AM. The fluorescence intensity was determined by a microplate fluorescence reader. The fluorescence was corrected by the background signal derived from nonmarked cells. All data are reported as *F*–*F*_0_ (*F* = fluorescence signal of BAECs stimulated with Bk; *F*_0_ = fluorescence signal of unstimulated BAECs) (a). Peak of cytosolic Ca^2+^ was reported as fold changes vs. controls, whose mean value was set as 1 (^∗∗^*p* < 0.01 vs. CTRL) (b). Plateau of Ca^2+^ kinetics in response to Bk was evaluated as area under the curve (AUC) of the plateau phase (starting from 100 seconds and up to 200 seconds after Bk administration). Data are reported as fold changes vs. controls, whose mean value was set as 1 (^∗∗^*p* < 0.01 vs. CTRL) (c). BAECs were treated with PTH (0.1 nM) for 24 hours, and NO release was assessed in response to Bk (30 nM), incubating the cells with the fluorescent probe DAF-FM Diacetate. The fluorescence intensity was determined by a microplate fluorescence reader. The fluorescence was corrected by the background signal derived from nonmarked cells. All data are reported as *F*–*F*_0_ (*F* = fluorescence signal of BAECs stimulated with Bk; *F*_0_ = fluorescence signal of unstimulated BAECs) (d). Peak of NO release was reported as fold changes vs. controls, whose mean value was set as 1 (^∗^*p* < 0.05 vs. CTRL) (e). All images are the mean of three independent experiments.

**Figure 2:**
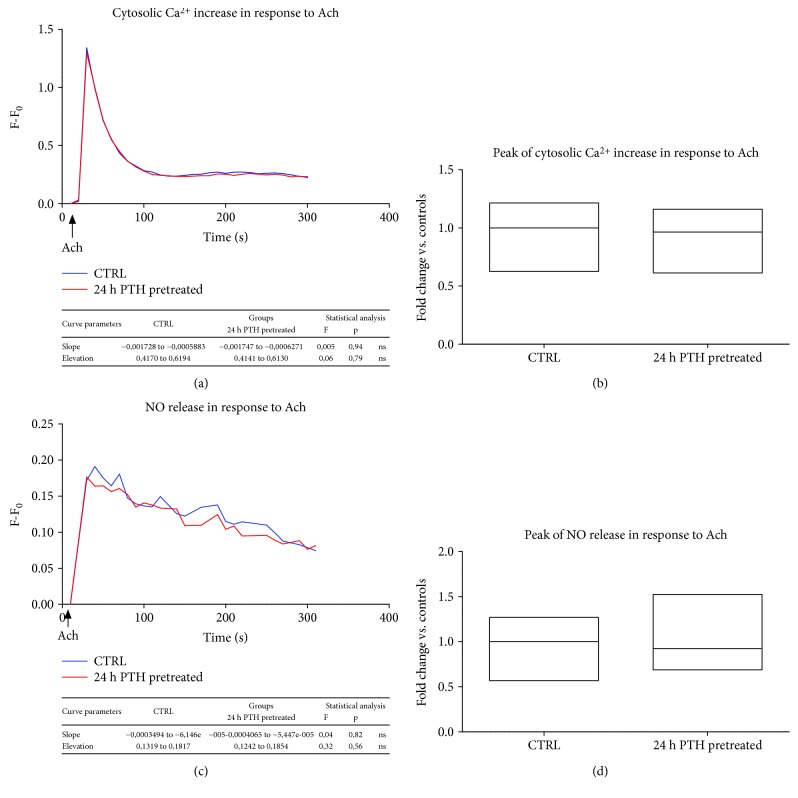
PTH spares endothelial responses to acetylcholine (Ach) (a–d). BAECs were treated with PTH (0.1 nM) for 24 hours, and Ca^2+^ fluxes were assessed in response to Ach (1 *μ*M), incubating the cells with the fluorescent probe Fluo4-AM. The fluorescence intensity was determined by a microplate fluorescence reader. The fluorescent signal was corrected for the background signal derived from nonmarked cells. All data are reported as *F*–*F*_0_ (*F* = fluorescence signal of BAECs stimulated with Ach; *F*_0_ = fluorescence signal of BAECs at basal condition) (a). Peak of cytosolic Ca^2+^ is reported as fold changes vs. controls, whose mean value was set as 1 (b). BAECs were treated with PTH (0.1 nM) for 24 hours, and NO release was assessed in response to Ach (1 *μ*M), incubating the cells with the fluorescent probe DAF-FM diacetate. The fluorescence intensity was determined by a microplate fluorescence reader. The fluorescence was corrected by the background signal derived from nonmarked cells. All data are reported as *F*–*F*_0_ (*F* = fluorescence signal of BAECs stimulated with Ach; *F*_0_ = fluorescence signal of unstimulated BAECs) (c). Peak of NO release was reported as fold changes vs. controls, whose mean value was set as 1 (d). All images are the mean of three independent experiments.

We explored PTH ability to compromise relevant signaling for endothelium homeostasis. Indeed, endothelial dysfunction is commonly defined as a deterioration of endothelium-dependent vasodilation, which is physiologically induced by several neurotransmitters and hormones through the production of NO [27]. Thus, we evaluated, *in vitro*, PTH ability to affect endothelial responsiveness to essential vasoactive mediators, such as bradykinin (Bk) and acetylcholine (Ach). In BAECs, cytosolic calcium accumulation and nitric oxide (NO) production were measured as markers of endothelial response (Figures 1 and 2). As expected, Bk (30 nM) acutely led to an increase in cytosolic calcium through a classic biphasic pattern, consisting in an initial Ca^2+^ peak followed by a plateau. The maximum cytosolic concentration was recorded 10 seconds after Bk exposure, while plateau was clearly evident after 100 seconds. Although PTH (0.1 nM–24 h) pretreatment did not determine any delay in Ca^2+^ peak or any modifications in the slope of Ca^2+^ kinetics (Figure 1(a)), it was able to reduce the overall Ca^2+^ response to Bk (Figure 1(a)). In particular, a 30.10% (95% CI 15.15%–45.04%, *p* < 0.01) reduction in cytosolic calcium maximum increase was found following PTH pretreatment (Figure 1(b)). Moreover, PTH reduced the amplitude of the Ca^2+^ plateau, assessed as area under the curve (AUC) from sec 100 to sec 200, by 46% (95% CI 33.19% to 59.17%, *p* < 0.01) (Figure 1(c)). Similarly, NO synthesis was stimulated by Bk exposure, as expected, and the concentration peak was recorded 40 seconds after the stimulus. NO synthesis is deeply influenced by Ca^2+^ [28], and store-operated Ca^2+^ entry (SOCE) represents a major regulator of eNOS activation in endothelial cells (recently reviewed in [29]). Likely, SOCE might contribute to the plateau phase of Bk-dependent Ca^2+^ kinetics, as it is activated by ER-Ca^2+^ emptying [30] represented by the peak of Ca^2+^ kinetics. According to the effects on plateau of Bk-dependent Ca^2+^ kinetics, PTH pretreatment (0.1 nM–24 h) severely affected also Bk-induced NO release (Figure 1(d)) causing an 85.72% (95% CI 2%–171%, *p* < 0.05) reduction of the maximum NO release (Figure 1(e)). No delay in the peak of NO release or any modifications in the slope of the kinetics were recorded (Figure 1(d)).

Also, Ach induced cytosolic Ca^2+^ fluxes and NO release, as expected. Opposite to Bk, both Ach-induced responses were not affected by PTH pretreatment (Figures 2(a)–2(c)). Indeed, no significant variations were measured between the control and 24 h pretreated BAECs in both peaks of Ca^2+^ increase and NO synthesis in response to Ach (Figures 2(b)–2(d)). These results suggested that PTH did not impair globally endothelial function but exerted a selective interference on endothelial responsiveness to Bk, preserving Ach effects.

### 3.2. PTH Acutely Induces Oxidative Stress

**Figure 3:**
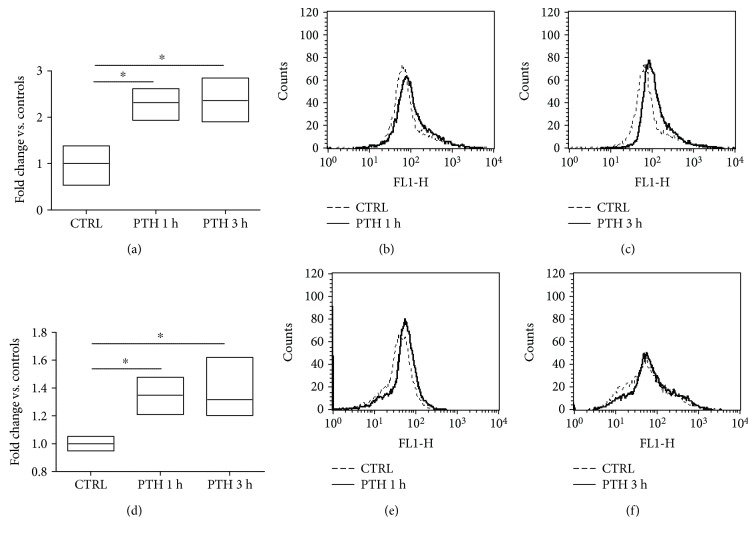
Evaluation of ROS production in response to PTH (a–f). BAECs were treated for 1 or 3 h with PTH (0.1 nM), and the total amount of ROS was determined incubating the cells with the fluorescent probe H_2_DCFDA. The fluorescence intensity was determined by citofluorimetry. The fluorescence relative to ROS levels was expressed as fold change vs. controls, whose mean value was set as 1 (^∗^*p* < 0.05 vs. CTRL). The image is the mean of three independent experiments (a). Representative images of flow cytometry of three independent experiments, evaluating tROS at 1 h (b) and 3 h (c) post-PTH treatment. The levels of mROS were determined in the same experimental condition, using Mitosox as specific fluorescent probe. The fluorescence relative to mROS levels was expressed as fold change vs. controls whose mean value was set as 1 (^∗^*p* < 0.05 vs. CTRL). The image is the mean of three independent experiments (d). Representative images of flow cytometry of three independent experiments, evaluating mitochondrial levels of ROS at 1 h (e) and 3 h (f) post-PTH treatment.

The key role of oxidative stress in endothelial dysfunction is well established [31]. In the endothelium, reactive oxygen species (ROS) overproduction can lead to lipids and protein oxidation, interfere with NO production, and impair response to vasodilators through other partially known mechanisms [32]. Therefore, we investigated whether PTH caused endothelial dysfunction through an increase in ROS production. BAECs were acutely exposed to PTH ((0.1 nM) 1–3 h), and both tROS and mROS levels were measured (Figure 3). PTH stimulation induced a significant increase in tROS production in all analyzed time points when compared to controls. In particular, we recorded a 1.35-fold increase (95% CI 0.5951 to 2.035, *p* < 0.01) (Figures 3(a) and 3(b)) and 1.362-fold increase (95% CI 0.6424 to 2.082, *p* < 0.01) (Figures 3(a)–3(c)) at 1 h and 3 h post-PTH stimulation, respectively. Interestingly, PTH also produced a significant accumulation of mROS at both 1 h [fold of increase, PTH vs. CTRL 0.348 (95% CI 0.0977 to 0.5998, *p* < 0.05)] (Figures 3(d) and 3(e)) and 3 h [fold of increase vs. CTRL 0.3167 (95% CI 0.0785 to 0.5548, *p* < 0.05)] (Figures 3(d)–3(f)). These results demonstrated that, after few hours from exposure, PTH was able to induce ROS overproduction, also involving mitochondria.

### 3.3. MitoTEMPO but not Vitamin D Attenuates PTH-Dependent mROS Production

**Figure 4:**
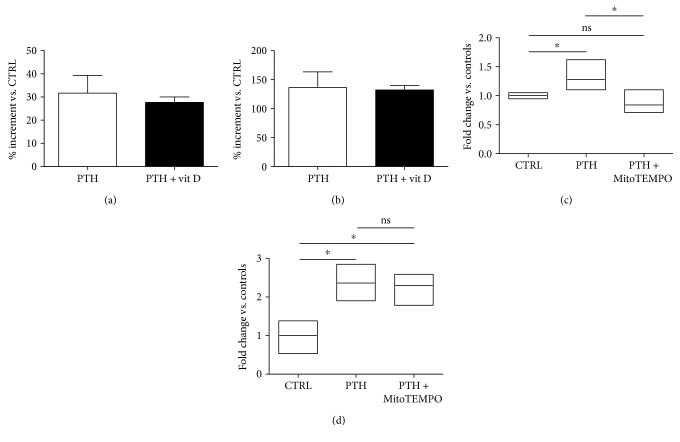
MitoTEMPO but not calcipotriol limits PTH-dependent ROS surge (a–d). The antioxidant properties of either calcipotriol or MitoTEMPO were tested on mitochondrial and total ROS productions in response to PTH (0.1 nM). BAECs were pretreated with calcipotriol (100 nM), stimulated with PTH at the same time, and as described above, mROS (a) and tROS (b) were determined. The data of fluorescence intensity were expressed as % increment vs. control and reported as mean ± SD. In another set of experiments, BAECs were pretreated with MitoTEMPO (5 *μ*M), stimulated 30 min later with PTH, and as described above, mROS (c) and tROS (d) were determined. The data of fluorescence intensity were expressed as fold change vs. control whose mean value was set as 1 (^∗^*p* < 0.05 vs. CTRL). All images are the mean of three independent experiments.

In order to evaluate whether PTH-dependent ROS production is responsible of the observed endothelial dysfunction, we performed the same experiments testing the endothelial responsiveness in the presence of two, well-established antioxidants, vitamin D [33] and MitoTEMPO, a specific mitochondria-targeted antioxidant [34]. As the first step, we verified the actual ability of both antioxidants to specifically inhibit ROS production in our experimental model, in response to PTH. As described in the introductive section, a functional crosstalk between PTH and calcitriol exists. Indeed, calcitriol is able to directly regulate the bioavailability of PTH. Since calcitriol displays antioxidant properties, we evaluated its ability to counteract ROS production induced by PTH in BAECs. As shown in Figure 4(a), the administration of calcipotriol hydrate, a vitamin D analogue, did not significantly affect mitochondrial ROS production mediated by PTH. The same result was obtained on total ROS production, with no significant difference in total ROS levels between cells exposed to PTH and cells exposed to PTH in the presence of calcipotriol (Figure 4(b)). These data allow to speculate that the weak calcipotriol antioxidant properties are not effective in neutralizing PTH-dependent ROS overproduction. Conversely, as shown in Figure 4(c), MitoTEMPO administration induced a significant decrease in PTH-dependent mitochondrial ROS production (PTH + MitoTEMPO: 0.842 ± 0.129 vs. PTH: 1.28 ± 0.072, *p* < 0.05), but it was not able to significantly affect total ROS production (PTH + MitoTEMPO: 2.297 ± 0.2567 vs. PTH: 2.36 ± 0.2725, *p* = ns) (Figure 4(d)), thus confirming its specificity on mROS targeting.

### 3.4. Attenuation of mROS Production by MitoTEMPO Prevents the Impairment of Bk Responsiveness Induced by PTH

**Figure 5:**
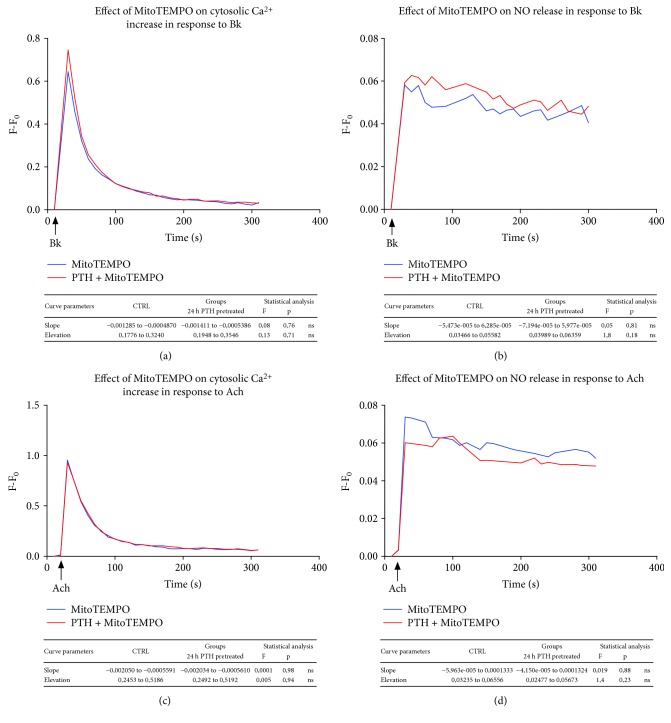
mROS scavenging with MitoTEMPO restores endothelial responses to Bk (a–d). In order to evaluate whether PTH-dependent ROS production is responsible of the endothelial dysfunction, we tested endothelial responsiveness to both Bk and Ach in the presence of a selective mROS scavenger (MitoTEMPO). BAECs were pretreated with MitoTEMPO (5 *μ*M), stimulated with PTH (0.1 nM) 30 minutes later and after 24 hours, both Ca^2+^ kinetics and NO release were assessed in response to either Bk (30 nM) (a–b) or Ach (1 *μ*M) (c–d), as described in Materials and Methods. The fluorescence intensity was determined by a microplate fluorescence reader. The fluorescence was corrected by the background signal derived from nonmarked cells. All data are reported as *F*–*F*_0_ (*F* = fluorescence signal of BAECs stimulated with either Bk or Ach; *F*_0_ = fluorescence signal of unstimulated BAECs). All images are the mean of three independent experiments.

Since MitoTEMPO (5 *μ*M) showed the ability to selectively scavenge PTH-dependent mitochondrial ROS, we tested its effect on endothelial responsiveness to Bk in endothelial cells pretreated with PTH (0.1 nM–24 h). In Figures 5(a) and 5(b), we showed that MitoTEMPO administration represented a very effective strategy to restore both cytosolic Ca^2+^ increase and NO synthesis evoked by Bk. Furthermore, we assessed the effects of MitoTEMPO on endothelial responsiveness to Ach in cells pretreated with PTH and found no relevant variations (Figures 5(c) and 5(d)). The lack of improvement in cytosolic Ca^2+^ increase and NO synthesis evoked by Ach suggested that MitoTEMPO exerted a selective effect on the pathways that have been compromised by PTH-induced mitochondrial ROS generation and did not act aspecifically, improving Ca^2+^ and NO-mediated responses of vasoactive mediators.

### 3.5. PTH Induces Oxidation of Bk Receptor B2, without Affecting mAch Receptor M1-M3 Oxidation

**Figure 6:**
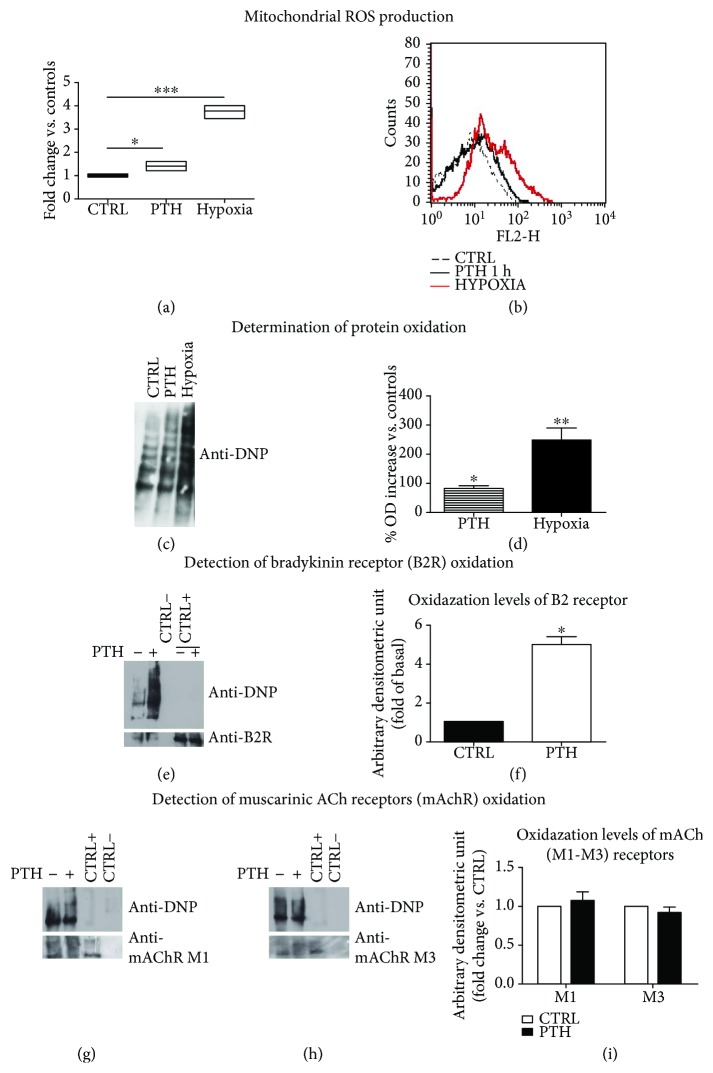
PTH selectively impairs Bk signaling through oxidation of Bk receptor B2 (a–i). BAECs were treated with PTH (0.1 nM) or exposed to hypoxia, and mROS levels were determined incubating the cells with the fluorescent probe Mitosox. The fluorescence intensity was determined by citofluorimetry. The fluorescence relative to ROS levels was expressed as fold change vs. controls (^∗^*p* < 0.05 vs. CTRL, ^∗∗∗^*p* < 0.0001 vs. CTRL). The image is the mean of three independent experiments (a). Representative images of flow cytometry of three independent experiments, evaluating mROS in response to PTH or hypoxia (b). The levels of protein oxidation in whole cell lysates of PTH pretreated or O_2_-deprived BAECs were determined by OxyBlot Protein Detection Kit as described in Materials and Methods and showed as levels of 2,4-dinitrophenylhydrazine (DNP) bound to proteins (c). Densitometric analysis of protein oxidation in response to either PTH or hypoxia is reported as mean % of increase ± SD vs. CTRL (^∗^*p* < 0.05 vs. CTRL, ^∗∗^*p* < 0.01 vs. CTRL) (d). The B2 receptor and mAch receptors (M1-M3) were immunoprecipitated from cells treated with PTH for 24 h, and the levels of receptor oxidation were determined by OxyBlot Protein Detection Kit as described in Materials and Methods and shown as levels of 2,4-dinitrophenylhydrazine (DNP) bound to the B2 receptor (e), mAch M1 receptor (g), and mAch M3 receptor (h). CTRL− represents an immunoprecipitation using the secondary antibody to test signal specificity. CTRL+ represents a whole cell lysate. The images are representative of results from three independent experiments. Densitometric analyses of the oxidized B2 receptor (f) and mAch M1-M3 receptors (i) are shown in bar graphs as mean ± SD (^∗^*p* < 0.05) of fold change vs. control. Both images are the mean of three independent experiments.

As we have shown above, PTH is able to alter endothelial responsiveness to BK in a mROS production–dependent manner. ROS are considered important signaling molecules, and their effects are often dependent on their intracellular levels. Indeed, to explain how the oxidative stress induced by PTH can alter selectively one specific pathway, we hypothesized that PTH-dependent ROS production does not compromise the global cellular responses. To test this hypothesis, we compared the mROS production induced by PTH to that observed in response to hypoxia, which is known to induce intensive mitochondrial ROS production. Although ROS production in response to PTH still occurs, it was significantly under the levels of mitochondrial ROS production induced by hypoxia [fold of increase PTH vs. CTRL: 0.348 (95% CI 0.0859 to 0.716, *p* < 0.05)/fold of increase hypoxia vs. CTRL: 2.770 (95% CI 2.411 to 3.129, *p* < 0.001)] (Figures 6(a) and 6(b)). The levels of protein oxidation also reflected this trend (Figures 6(c) and 6(d)). Interestingly, PTH exposure was able to induce oxidation of the BK receptor, B2 (Figures 6(e) and 6(f)). This posttranslational modification of the receptor might drive the dysfunctional responsiveness to BK induced by PTH. Conversely, PTH exposure did not affect the oxidation levels of muscarinic Ach receptors M1-M3 (mAchR M1-M3) explaining the preserved responsiveness of endothelial cells to Ach (Figures 6(g)–6(i)). Overall, these data suggest that ROS induced by PTH act as a signaling modulator altering specific pathways and preserving others.

### 3.6. PTH-Induced Production of Mitochondrial ROS and the Resulting Endothelial Dysfunction Are Driven by the Uptake of Calcium in the Mitochondria

**Figure 7:**
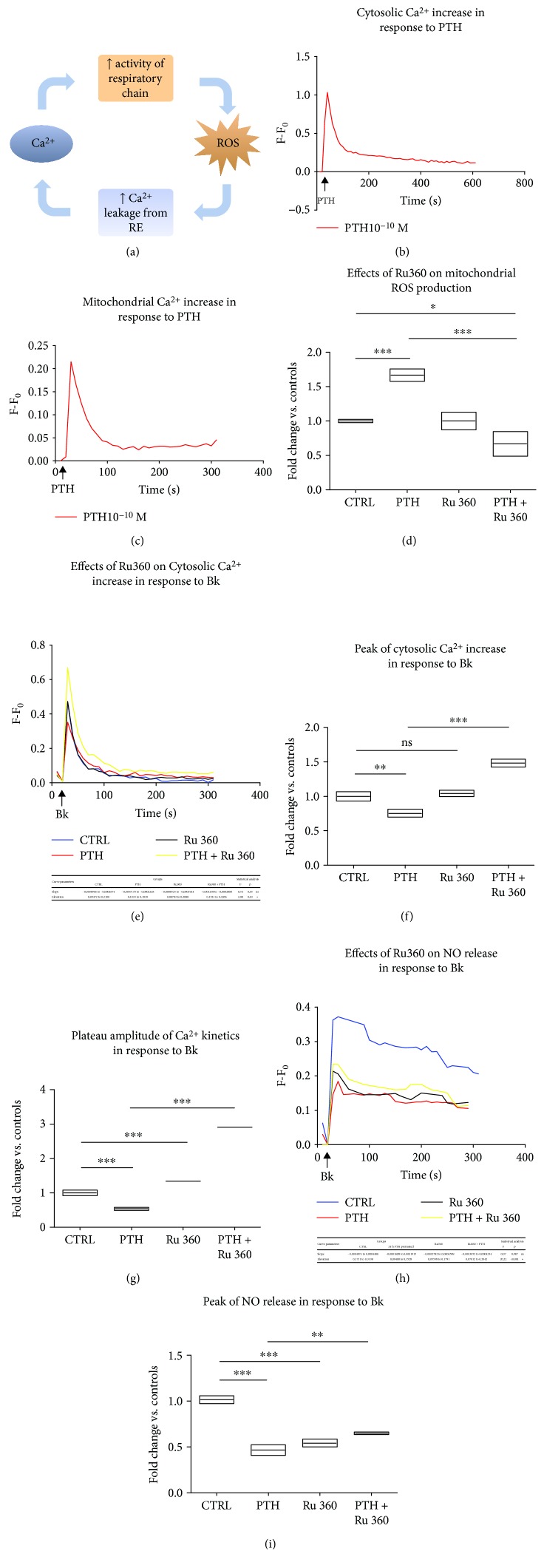
The inhibition of mitochondrial Ca^2+^ uptake limits PTH-dependent mitochondrial ROS production and endothelial dysfunction (a–i). The functional interplay between Ca^2+^ signaling and ROS production is a well-established concept. Briefly, Ca^2+^ released from intracellular stores is partially uptaken through MCU by mitochondria where it stimulates the respiratory chain. As soon as mitochondria are overloaded with calcium, the higher rate of oxygen consumption results in increased respiratory chain electron leakage and mitochondrial ROS production. In turn, ROS further affects Ca^2+^ fluxes across membranes through the oxidation of key regulators of Ca^2+^ homeostasis, such as ryanodine receptors (RyR) and sarco-endoplasmic reticulum Ca^2+^ ATPase (SERCA) (a). BAECs were acutely stimulated with PTH (0.1 nM), and both cytosolic (b) and mitochondrial (c) Ca^2+^ were evaluated with specific fluorescent probes, Fluo4-AM and Rhod-2 AM, respectively, through a microplate fluorescence reader. The fluorescence was corrected by the background signal derived from nonmarked cells. All data are reported as *F*–*F*_0_ (*F* = fluorescence signal of BAECs stimulated with PTH; *F*_0_ = fluorescence signal of unstimulated BAECs). In another set of experiments, BAECs were pretreated with Ru360 (10 *μ*M), stimulated with PTH (0.1 nM), and as described above, mROS levels were determined. The data of fluorescence intensity were expressed as fold change vs. control whose mean value was set as 1 (^∗∗∗^*p* < 0.001) (d). BAECs were pretreated with Ru360 (10 *μ*M), then stimulated with PTH (0.1 nM) for 24 hours, and Ca^2+^ fluxes were assessed in response to Bk (30 nM), as described above. All data are reported as *F*–*F*_0_ (*F* = fluorescence signal of BAECs stimulated with Bk; *F*_0_ = fluorescence signal of unstimulated BAECs) (e). Peak of cytosolic Ca^2+^ increase was reported as fold changes vs. control, whose mean value was set as 1 (^∗∗∗^*p* < 0.001) (f). Plateau amplitude of Ca^2+^ kinetics in response to Bk was evaluated as area under the curve (AUC) of the plateau phase (starting from 100 seconds after Bk administration). Data are reported as fold changes vs. control, whose mean value was set as 1 (^∗∗∗^*p* < 0.001) (g). BAECs were pretreated with Ru360 (10 *μ*M), then stimulated with PTH (0.1 nM) for 24 hours, and NO release was assessed in response to Bk (30 nM), as described above. All data are reported as *F*–*F*_0_ (*F* = fluorescence signal of BAECs stimulated with Bk; *F*_0_ = fluorescence signal of unstimulated BAECs) (h). Peak of NO release was reported as fold changes vs. control, whose mean value was set as 1 (^∗∗^*p* < 0.01) (i). All images are representative of three independent experiments.

PTH exerts its metabolic effects on target cells by binding a G protein-coupled receptor (GPCR) named PTH1 receptor (PTH1R), which couples both to the adenylyl cyclase– (AC–) protein kinase A (PKA) signaling pathway and to the phospholipase C– (PLC–) protein kinase C- (PKC-) intracellular Ca^2+^ signaling pathway [35]. Thus, cytosolic Ca^2+^ represents a key signaling also in cellular responses to PTH. In order to explore the mechanisms by which PTH induced mitochondrial ROS production, we evaluated whether PTH is able to recruit the functional interplay between Ca^2+^ and ROS, described elsewhere (Figure 7(a)) [36]. As expected, PTH promoted Ca^2+^ mobilization in the cytosol, and the maximum concentration was recorded 10 seconds after the stimulus (Figure 7(b)). Interestingly, PTH was also able to induce Ca^2+^ uptake in the mitochondria very quickly (10 seconds, as cytosolic Ca^2+^) (Figure 7(c)), suggesting that at least part of cytosolic Ca^2+^, which is released from intracellular stores in response to PTH, is shunted to these organelles to modulate still unknown functions. In order to assess the involvement of PTH-induced mitochondrial Ca^2+^ fluxes in the mitochondrial ROS generation, we evaluated the effect of Ru360 (10 *μ*M), a mitochondrial calcium uniporter (MCU) inhibitor, [37] on basal and PTH-induced mitochondrial ROS productions. Ru360 did not influence mitochondrial ROS levels at basal conditions but significantly prevented mitochondrial ROS generation evoked by PTH (0.1 nM). In particular, we recorded a 0.99-fold (95% CI 0.6718 to 1326, *p* < 0.0001) of decrease in mitochondrial ROS generation when BAECs were treated with Ru360 (Figure 7(d)). Since we have shown above that MitoTEMPO exerted a preventive effect on PTH-induced dysfunctional endothelial responsiveness to Bk through the scavenging of mitochondrial ROS, we evaluated whether Ru360 was able to reproduce the same results by preventing mitochondrial ROS generation. As shown in Figure 7(e), we found an improvement of cytosolic Ca^2+^ increase in response to Bk when PTH-pretreated BAECs were incubated with Ru360, suggesting that the latter might prevent the dysfunctional phenotype induced by PTH. In particular, a 73.10% (95% CI 58.15%–88.05%, *p* < 0.001) improvement in cytosolic calcium maximum increase was found in Ru360 + PTH–treated cells when compared to PTH alone–treated BAECs (Figure 7(f)). Moreover, Ru360 in PTH-pretreated cells improved even plateau amplitude of Ca^2+^ kinetics, assessed as area under the curve (AUC) of the plateau phase, by 2.37-fold (95% CI 2.27 to 2.46, *p* < 0.001) (Figure 7(g)). Interestingly, Ru360 alone improved Ca^2+^ amplitude of the plateau phase by 33.81% when compared to control BAECs (95% CI 24.32% to 43.31%, *p* < 0.001 vs. CTRL). Likely, as mitochondria shape ER-dependent Ca^2+^ release [38], Ru360 prevented mitochondrial buffering of Ca^2+^ leading to a transient cytosolic retention of Ca^2+^ in the plateau phase. However, in PTH-pretreated cells, Ru360-mediated increase of cytosolic Ca^2+^ was much more pronounced suggesting that inhibition of the ROS surge was actually the most relevant mechanism involved (Figure 7(g)). Ru360 improved also NO release, which has been impaired by PTH pretreatment, although it was unable to fully restore physiological levels of Bk-dependent NO production (Figures 7(h) and 7(i)). The partial recovery of NO release might be justified by the finding that Ru360 alone decreased NO production when compared to control BAECs, more likely through the inhibition of mitochondrial nitric oxide synthase (mtNOS), as described elsewhere [39].

### 3.7. PTH Chronic Exposure Reduces Angiogenic Competence of Endothelial Cells *In Vitro*

**Figure 8:**
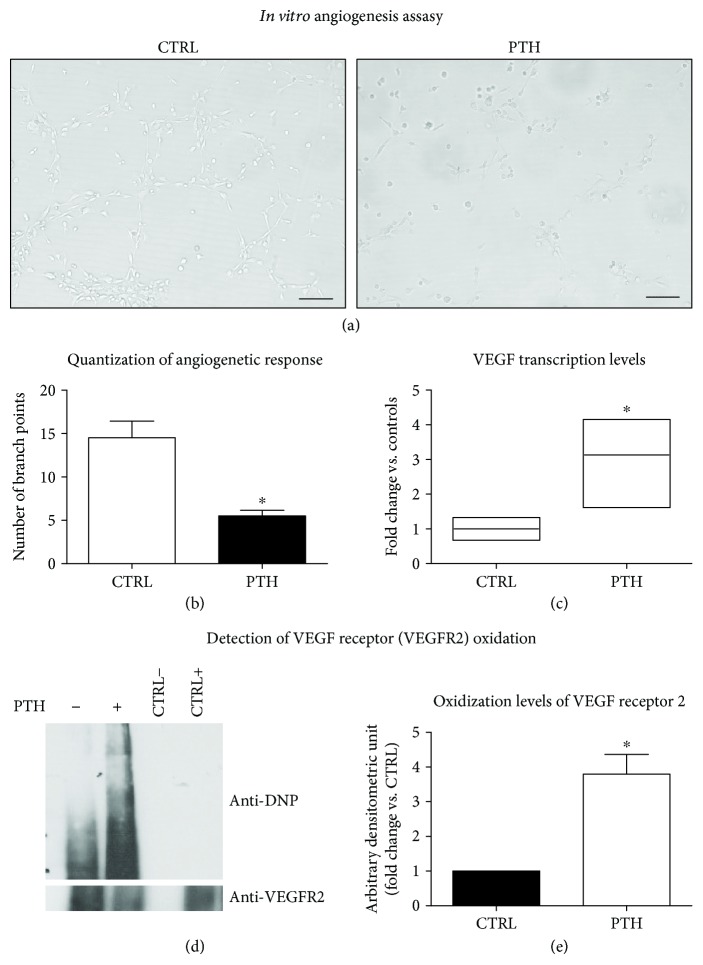
Characterization of angiogenic phenotype in response to PTH (a–e). BAECs were plated on Matrigel matrix, and tubular formations were evaluated 24 h after PTH (0.1 nM) treatment and compared to control. Pictures of tubular formations are obtained using an optical microscope; the images are representative of results from three independent experiments. The scale bar is about 20 *μ*m (a). The angiogenic response was quantized as the number of branch points, and the data are reported as mean ± SD (^∗^*p* < 0.05 vs. CTRL) (b). From BAECs, after 24 h of PTH treatment, mRNA was isolated; the transcription levels of VEGF were evaluated by real-time PCR and expressed as fold increase vs. control (^∗^*p* < 0.05 vs. CTRL) (c). VEGF receptor was immunoprecipitated from cells treated with PTH for 24 h. The levels of VEGF receptor oxidation were determined by OxyBlot Protein Detection Kit as described in Materials and Methods and shown as levels of 2,4-dinitrophenylhydrazine (DNP) bound to the receptor. CTRL− represents an immunoprecipitation using the secondary antibody to test signal specificity. CTRL+ represents a whole cell lysate. The images are representative of results from three independent experiments (d). Densitometric analysis of oxidized VEGFR2 is shown in the bar graph as mean ± SD (^∗^*p* < 0.05) of fold change vs. control (e). The image is the mean of three independent experiments.

To verify more complex phenotypes of PTH-induced endothelial dysfunction, we evaluated angiogenic responses *in vitro*. BAECs were seeded on Matrigel, and after PTH treatment (0.1 nM–24 h), we assessed tubular formations. Matrigel induced the classical tubular network organization of BAECs after 24 h. PTH induced a visible alteration of cell contacts needed for tubular formations (Figure 8(a)), quantified as significant reduction of the branch point number (PTH: 5.50 ± 0.64 vs. CTRL: 14.50 ± 1.93) (Figure 8(b)), thus demonstrating that PTH exposure impairs angiogenesis *in vitro*. In order to evaluate whether the compromised angiogenesis was due to reduction in vascular endothelial growth factor (VEGF) production, we determined VEGF transcriptional levels after PTH exposure. Interestingly, PTH chronic treatment induced an increase in VEGF gene transcription (Figure 8(c)), thus suggesting that PTH might have altered angiogenic competence inducing resistance to VEGF, a common feature of endothelial dysfunction. Since PTH was able to induce selective signaling impairment through posttranslational oxidative modification of signaling proteins, we evaluated the levels of VEGF receptor oxidation. The exposure to PTH induced a significant increase of VEGF receptor oxidation (Figures 8(d) and 8(e)), suggesting that this posttranslational modification might be responsible of both altered VEGF-dependent signal transduction and impaired angiogenic competence.

## 4. Discussion

Altogether, our data represent the first direct evidence that PTH causes endothelial dysfunction in a ROS-dependent manner, through a surge of mitochondrial Ca^2+^. This oxidative response leads to the impairment of selected signal transduction pathways, with altered NO production and impaired endothelial phenotypes such as angiogenesis.

Cardiac tissue and the vascular system express the PTH receptor, and the putative role of PTH in the regulation of myocardial function and vasculature homeostasis has inspired several researches in the last years. During heart failure, a calcium deprivation occurs inducing PTH release, and the increased levels of the hormone could contribute to maladaptive responses [21, 23, 40], bearing important clinical and therapeutic implications. Beyond PTH detrimental effects on the myocardium [41, 42], several studies have also explored the impact of hyperparathyroidism on vascular homeostasis. Indeed, it was hypothesized that PTH-mediated bone-renal-vascular interactions might exert a pivotal role in PTH-dependent increased CVR [14]. The PTH role in a broad spectrum of vascular disorders such as coronary microvascular dysfunction, vascular calcification, increased vascular stiffness, and blood pressure is a well-established concept, in both primary and secondary hyperparathyroidisms [43, 44]. Mounting evidence suggests that endothelium might be a key target of PTH. Indeed, hyperparathyroidism correlates with clinical biomarkers of impaired endothelial function, such as flow-mediated dilation and intima-media thickness [45–48], which can be reversed by parathyroidectomy [49]. However, direct evidence of PTH-induced endothelial dysfunction is missing, as well as the understanding of the underlying mechanisms. Therefore, our study is innovative in the fact that for the first time we show PTH-induced endothelial dysfunction in terms of responsiveness to vasodilators and angiogenic competence. The impairment of endothelium-dependent relaxation represents a key feature of endothelial dysfunction found in many cardiovascular diseases, such as hypertension and heart failure [50].

Among the candidate mechanisms able to induce endothelial dysfunction, we chose to investigate the role of ROS since the presence in literature of indirect evidence about the relation between PTH levels and markers of oxidative stress [22, 23]. We found that PTH induces mitochondrial ROS production in a calcium-dependent manner. Using selective inhibitors of the MCU, we provide the first ever evidence that mitochondrial calcium accumulation is the first causative molecular event that drives the observed biological effects of PTH on endothelial cells.

PTH-induced ROS production is about 2-folds, well below the ROS production induced by a massive stress, like hypoxia. Likely, the ROS production in response to PTH is at levels that make it a signaling modifier [51, 52], rather than producing global and not specific cellular alterations. This is even more so when considering that PTH selectively attenuates Bk-dependent but not Ach NO release. We show that Bk receptor (B2) but not muscarinic receptors M1 and M3 represents a target of PTH-dependent oxidative stress being significantly oxidized by PTH-induced ROS surge. Such oxidation, by altering the receptor conformation or the availability of functional residues, could compromise the Bk signaling transduction, thus explaining the reduced responsiveness to Bk.

Our data show that the exposure of endothelial cells to PTH induced a significant reduction of angiogenetic competence *in vitro*. This result confirms and exploits previous suggestions of a putative impact of PTH on angiogenesis gathered in rats with TAC-induced heart failure [41]. In our model, the impairment of angiogenesis occurred despite an increase of VEGF mRNA. This paradoxical response is further supported by previous literature showing that *in vitro*, PTH induces an increase of VEGF mRNA without affecting its protein levels [53]. The missed angiogenetic response can be alternatively justified by VEGF resistance, a recently described feature of dysfunctional endothelium [32]. Our data that VEGF receptor oxidative modifications induced by PTH mediated the ROS surge offers a conciliant molecular explanation to the putative dysfunctional phenotype of VEGF resistance.

Ca^2+^ represents one of the most common signal transduction molecules known. Indeed, several stimuli trigger Ca^2+^ release from intracellular stores to control a wide array of biological functions including muscle contraction, synaptic transmission, hormone secretion, excitability, and programmed cell death [54]. PTH ability to activate dual signaling poses the question whether Ca^2+^ rather than cAMP is responsible for the observed PTH-induced endothelial cell dysfunction. Our data support this hypothesis and in particular the role of mitochondrial Ca^2+^ to trigger ROS activation.

**Figure 9:**
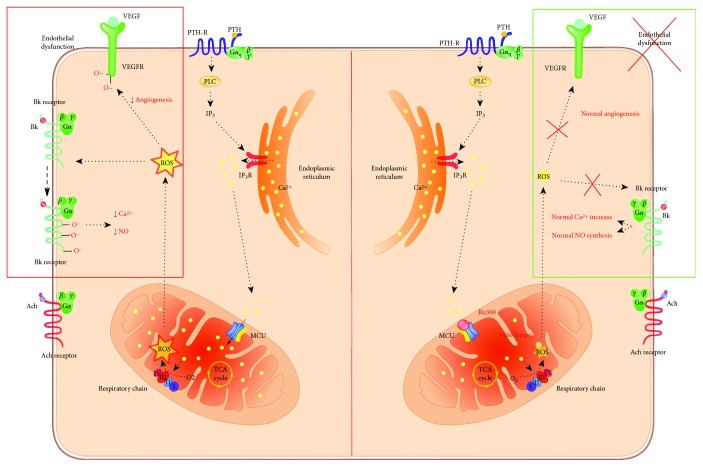
PTH-dependent endothelial dysfunction: hypothesis of molecular mechanisms involved. PTH, through IP_3_ production, induces increase of cytosolic calcium with relative increase in mitochondrial calcium uptake by MCU. The resultant mitochondrial calcium overload determines an increase of ROS production which in turn mediates the alteration of certain signaling (Bk, VEGFR) rather than other ones (Ach) by protein oxidation thus perturbing the endothelial functionality. Ru360 and MitoTEMPO, respectively, by inhibiting MCU and scavenging mitochondrial ROS, prevent perturbations in key signaling molecules for endothelial homeostasis.

Overall, these data allow us to provide a model of molecular mechanism underlying the action of PTH, summarized in Figure 9.

**Figure 10:**
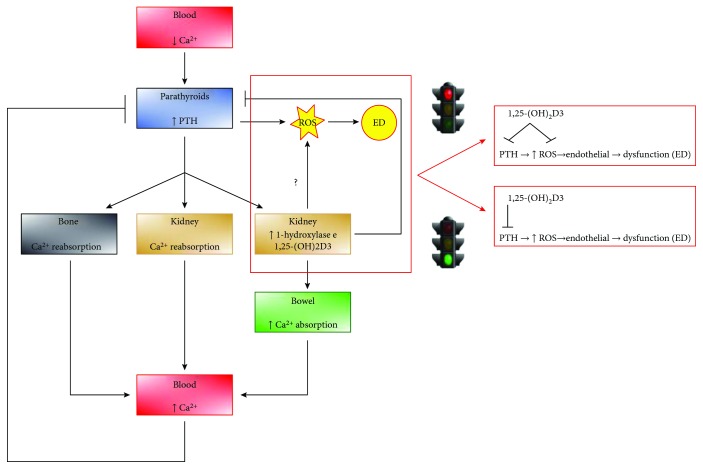
Vitamin D supplementation putatively reduces CVR by lowering PTH serum levels. Since vitamin D exposure failed to lower PTH-dependent ROS production, we can assume that the correction of CVR depends on PTH serum level normalization. This effect derives from both PTH gene downregulation by vitamin D/vitamin D intracellular receptor complex and increased blood Ca^2+^ concentrations.

Our data, by putting in relationship PTH and endothelial dysfunction, a mechanism of CVR, provide a possible explanation of the discordant observations regarding the increased CVR in vitamin D–deprived populations and the lack of efficacy of clinical trials based on vitamin D supplementation [55]. According to our vision, the CVR would increase only among those patients in which the vitamin D insufficiency leads to increased serum PTH [14]. Consequently, vitamin D supplementation would be effective in reducing CVR only among those patients where (1) PTH is increased due to vitamin D insufficiency and (2) PTH is normalized after vitamin D supplementation [56]. Although specific clinical or experimental trials are needed to better clarify this issue, our data support this vision. Indeed, vitamin D supplementation can putatively ameliorate endothelial dysfunction induced by PTH, either by its antioxidative properties or by reduction of serum PTH levels over time. In fact, vitamin D exposure in our model failed to acutely reduce PTH-induced ROS production, ruling out the possibility that vitamin D can restore endothelial dysfunction due to its weak antioxidant properties. Accordingly, we can speculate that the success of vitamin D supplementation on CVR lowering needs preventively a stratification of patients with vitamin D deficiency according to PTH plasma levels. The coexistence of hyperparathyroidism and vitamin D deficit might identify the clinical feature of patients that could more likely take advantage of the therapy.  Figure 10 summarizes these hypotheses.

### 4.1. Future Perspectives

*In vivo* experiments are needed to further confirm the determinant role of PTH, instead of vitamin D deficiency, in endothelial dysfunction. A mouse model of vitamin D resistance–induced hyperparathyroidism treated with blocker of the PTH receptor might account for this purpose. Moreover, clinical trials with vitamin D supplementation in patients with vitamin D deficiency stratified according to PTH levels should be performed to test our hypothesis.

## Data Availability

The data used to support the findings of this study are included within the article.
